# Supplementary material for: Thymopentin Enhances Antitumor Immunity Through Thymic Rejuvenation and T Cell Functional Reprogramming
Source: Biomedicines. 2025 Oct 13;13(10):2494. doi: 10.3390/biomedicines13102494 (PMC12561324; doi:10.3390/biomedicines13102494)
Supplement: Supplementary file 1 [file biomedicines-13-02494-s001.zip › biomedicines-3857278-supplementary.pdf]

Supplementary material

Supplementary Figure S1

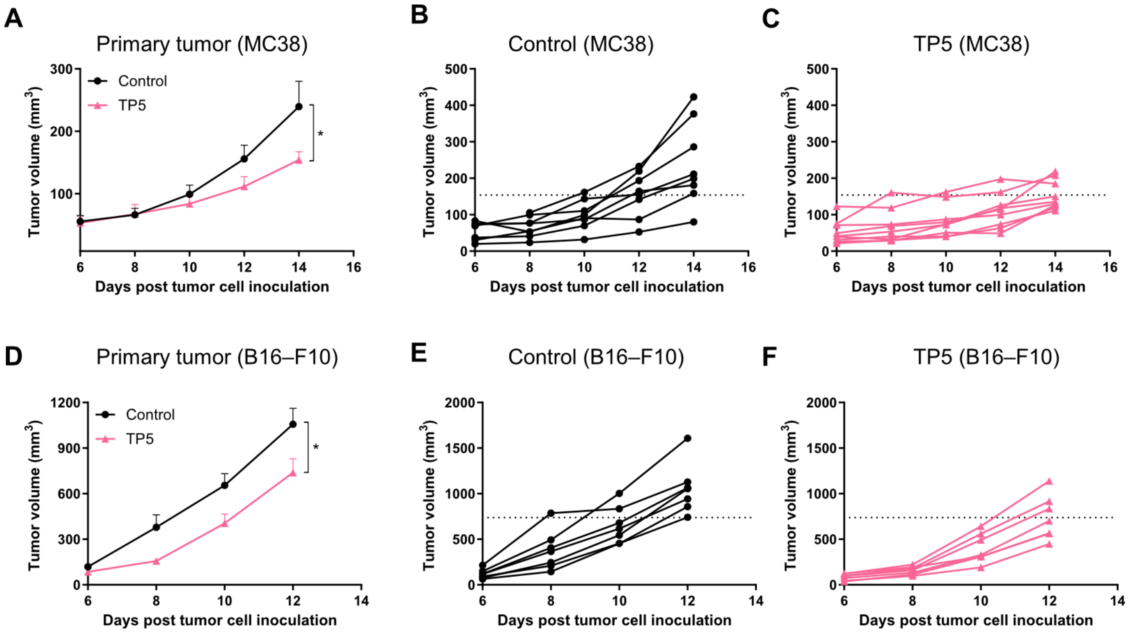

Supplementary Figure S1. TP5 inhibited tumor growth in mice. (A-C) C57BL/6 mice were inoculated with MC38 (1×10^6 cells/mouse) to generate subcutaneous colon carcinoma and treated with TP5 (20mg/kg/day) or saline from day 6 to day 14: (A) tumor progression of MC38 model; (B) individual tumor growth curve of control mice; (C) individual tumor growth curve of TP5 treated mice (n=8 mouse per group for a-c). (D-F) C57BL/6 mice were inoculated with B16-F10 (5×10^5 cells/mouse) to generate subcutaneous melanoma and treated with TP5 (20mg/kg/day) or saline from day 6 to day 12: (D) tumor progression of B16-F10 model; (E) individual tumor growth curve of control mice; (F) individual tumor growth curve of TP5 treated mice (n=7 mouse per group for d-f). The lines in graphs (B,C,F,G) are based on the average tumor volume of TP5 group at the endpoints for each experiments. p values were calculated using ordinary two-way analysis of variance (ANOVA) for (A,D). Data presented as mean ± SEM.* $p < 0.05$.
